# Properties of a Glutamatergic Synapse Controlling Information Output from Retinal Bipolar Cells

**DOI:** 10.1371/journal.pone.0129133

**Published:** 2015-06-08

**Authors:** Santhosh Sethuramanujam, Malcolm M. Slaughter

**Affiliations:** Center for Neuroscience and Department of Physiology and Biophysics, University at Buffalo, Buffalo, New York, United States of America; The University of Melbourne, AUSTRALIA

## Abstract

One general categorization of retinal ganglion cells is to segregate them into tonically or phasically responding neurons, each conveying discrete aspects of the visual scene. Although best identified in the output signals of the retina, this distinction is initiated at the first synapse: between photoreceptors and the dendrites of bipolar cells. In this study we found that the output synapses of bipolar cells also contribute to separate these pathways. Both transient and sustained ganglion cells can produce maintained spike activity, but bipolar cell glutamate release exhibits a divergence that corresponds to the response characteristics of the ganglion cells. Comparing light intensity coding in the sustained and transient ON pathways revealed that they shared the intensity spectrum. The transient pathway had greater sensitivity but smaller dynamic range, and switched from intensity coding to event detection at light levels where sustained pathway sensitivity began to rise. The distinctive properties of the sustained pathway depended upon inhibition and shifted toward those of the transient pathway in the absence of inhibition. The transient system was comparatively unaffected by the loss of inhibition and this was due to the concomitant activation of perisynaptic NMDA receptors. Overall, the properties of bipolar cell dendritic and axon terminals both contribute to the formation of key aspects of the sustained/transient dichotomy normally associated with ganglion cells.

## Introduction

Bipolar cells are the interlocutor between the light transduction of photoreceptors and the neural code of ganglion cells. Much of their information content is determined by transformations that occur at the junction with rods and cones. At this synapse it is the properties of the postsynaptic bipolar cells that establish the parallel ON-OFF [1] and transient-sustained pathways [2, 3], fundamental circuits throughout the central nervous system. At the next synapse the bipolar cell signals are shaped by inhibitory feedback [4–7], contributing to complex feature extraction such as edge detection or directional selectivity [8–10].

This second synapse often connects bipolar cells to both amacrine and ganglion cells. The amacrine cells provide both feedback and feedforward inhibition. In vertebrate retina there are about a dozen bipolar cell subtypes but over 20 categories of ganglion cells [11–13]. This disparity reflects complex processing that occurs as signals are transferred from bipolar cells to ganglion cells [4].

The picture that emerges is that the properties formed at bipolar cell dendrites are then modified at the axon terminal by amacrine cell feedback. However, in this study we asked whether the fundamental separation of information initiated at bipolar cell dendrites is reinforced at the output synapse of the bipolar cell. To do this we focused on the glutamate signal generated by sustained and transient pathways.

The results indicate that both sustained and transient ganglion cells are capable of tonic spike firing but that the release properties of bipolar cells differ in these two pathways. Furthermore, perisynaptic NMDA receptor (NMDAR) expression is similar in these pathways, but inhibitory regulation of their activation differs. Sustained and transient cells divide the intensity coding space. Inhibition is critical in this coding by sustained cells, while NMDARs compensate for a loss of inhibition in transient cells.

## Methods

### Tissue preparation

Larval tiger salamanders (*Ambystoma tigrinum*) were obtained from Charles Sullivan (Nashville, TN) and Kons Scientific (Germantown, WI) and were kept in tanks maintained at 4°C on a 12 h light/dark cycle. The animals were decapitated, and the eyes were enucleated. All procedures were performed in accordance with the US Animal Welfare Act and the National Institutes of Health’s Guide for the Care and Use of Laboratory Animals and were approved by the University Animal Care Committee at the State University of New York. The methods have been described in detail elsewhere [14]. Briefly, the retina was isolated in oxygenated Ringer’s solution under infrared light and mounted on a glass coverslip (Bellco Glass, Vineland, NJ). For slices, the retina was flat mounted ganglion side up on a 0.22 μm pore membrane filters (Millipore, Bedford, MA) and sliced at 150–250 μm using a tissue slicer (Stoelting, Woods Dale, IL). All electrophysiological experiments were done under infrared light. An infrared sensitive CCD camera (Hamamatsu, Japan) was used to display the image on an external monitor for viewing.

The tissue was constantly superfused with oxygenated Ringer’s solution containing (in mM): 111 NaCl, 2.5 KCl, 1.8 CaCl_2_, 1 MgCl_2_, 5 HEPES and 10 dextrose buffered to pH 7.8 using NaOH. A gravity-fed perfusion system was used to maintain a flow rate of ~1.5 ml/min.

### Electrophysiology

Recordings were made from neurons in the ganglion cell layer of both wholemounts and slices at room temperature. Extracellular spike activity was measured with a loose seal (25–50 MΩ) using an 8–10 MΩ electrode filled with Ringer’s solution. Based on the extracellular spike recordings, identified ON-OFF transient cells and ON sustained cells were patched for whole cell recordings using a separate, 5–7 MΩ electrode containing (in mM): 100 potassium gluconate, 5 NaCl, 1 MgCl_2_, 5 HEPES, 5 EGTA buffered to pH 7.4 with KOH.

Neurons were tentatively identified as ganglion cells based on their presence in the ganglion cell layer. The selected neurons had relatively large somas and produced multiple spikes, as opposed to other cells with smaller somas that produced either no spikes or only 1–2 spikes, and were tentatively identified as displaced amacrine cells. However, the neurons were not stained and therefore not positively identified. Hence, the records are described as coming from ganglion layer cells (GLCs), or as sustained or transient cells.

Data were acquired using a Multiclamp 700B Amplifier (Molecular Devices, Sunnyvale, CA). Analog signals were low-pass filtered at 2 kHz and sampled at 10 kHz with the Digidata 1322A analog-to-digital board (Molecular devices). Clampex 10.1 software (Molecular Devices) was used to control the voltage command outputs, acquire data and trigger stimuli. The currents and voltages shown are raw data and were not corrected for electrode junction potential and access resistance. Drug solutions were delivered through a pressure fed Octaflow 2 perfusion system (ALA Scientific Instruments, Farmingdale, NY). Picrotoxin and strychnine were purchased from Sigma-Aldrich Corp. AP5 (D-2-amino-5 phosphonovaleric acid), TBOA (DL-*threo*-β-benzyloxyaspartic acid) and cyclothiazide (6-chloro-3,4-dihydro-3-(5-norbornen-2-yl)-2*H*-1,2,4-benzothiazidiazine-7-sulfonamide-1,1-dioxide)were obtained from Tocris Bioscience (Minneapolis, MN).

### Stimulation protocol

#### Light stimulation

Photoreceptors were stimulated by a 200 μm spot from a red light-emitting diode (LED, λ_max_ = 640 nm) projected through the objective lens. The irradiance was calculated between 620 and 660 nm of the LED in μW cm^-2^, measured by a RPS900-R wideband spectroradiometer (International light, Peabody, MA). The light intensity was converted to photons/μm^2^/s. The light intensity was varied between 0.7–2734 photons/μm^2^/s. A one second light stimulus was presented every 25 seconds.

#### Electrical stimulation

Bipolar cells in retinal slices were directly stimulated by short pulses (1 ms) of current delivered through an electrode filled with Ringer’s solution that was placed directly into the outer plexiform layer above the patched GLC [15]. The pulses were generated with a constant-current stimulator (Grass S48 with stimulus isolation unit PSIU6, Grass Instruments, West Warwick, RI).

### Data analysis

Responses were plotted against light intensity, the data points were fitted with the Naka-Rushton equation [16];

$$R=R_{max}\left(\frac{I^{N}}{I^{N}+\sigma^{N}}\right)$$ (1)

where *R* indicates the response at a given intensity *I*, *R*_*max*_ indicates the maximum response, *σ* indicates the light intensity which produces a half-maximal response and *N*,a constant. Later work [17] proposed that the dynamic range or intensity span of the cell can be determined as the range of intensities that produce a response between 5% and 95% of *R*_*max*_ and can be calculated by

$$Intensity\;span=\frac{2.56}{N}$$ (2)

where *N* is the value from Eq (1). The light intensity inducing 50% of R_*max*_ (half maximum) was considered a measure of sensitivity.

Traces were imported into IgorPro 6.22 (Wavemetrics, Inc.) and Clampfit 10.1 (Molecular Devices) for making figures and further analysis. The Naka-Rushton fits to intensity-response relationships were constrained to R_max_ = 1. The fits were obtained using IgorPro’s algorithm for least-square data fitting. The total charge transfer by the EPSC for the duration of the light stimulus was used as a measure of the ON light response. The pooled data were imported to Microsoft Excel to make graphs and for statistical tests. Pooled data are expressed as mean ± standard error. Student’s t-test was used to compare values in different conditions, and was unpaired only when data were compared between transient and sustained responses. Differences were considered significant when p ≤ 0.05.

## Results

Recordings were made from neurons in the ganglion cell layer of the wholemount isolated salamander retina. ON sustained and ON-OFF transient cells were identified by their light-evoked spike activity in response to a 1s light stimulus using the loose patch recording technique. Then whole cell patch recordings were obtained from the identified cells, using either voltage or current clamp techniques.

### Transient cells can produce sustained spiking

**Fig 1:**
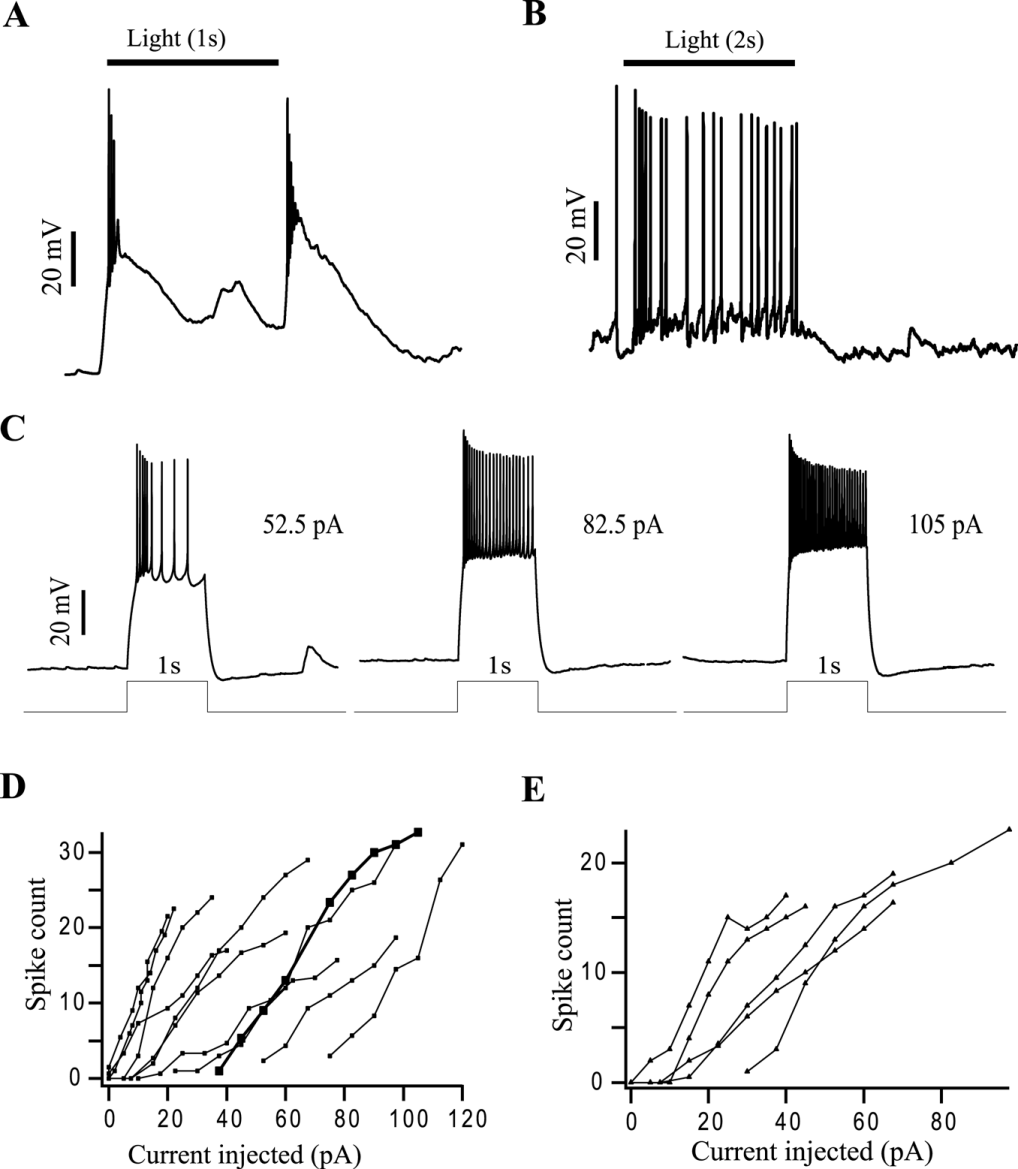
Both transient and sustained GLCs can produce sustained spiking. (A) and (B**)** show light evoked EPSPs and spike activity of a transient and sustained cell, respectively. The duration of light is shown by the black bar on top. (C) shows the voltage response of the transient cell shown in (A) to constant current injection of three different strengths. The duration of the current is shown by the step below the voltage responses. (D) shows a plot of the number of spikes evoked vs. injected current amplitude in 10 transient ON-OFF cells. The bold trace represents the cell shown in (C). (E**)** shows a plot of number of spikes evoked vs. injected current amplitude in 5 sustained ON cells.

An initial question was whether sustained and transient responding ganglion cells had distinct membrane properties that were responsible for their tonic and phasic spike patterns. Studies in isolated cells of fish and salamander retina indicate that some cells spike transiently while others respond in a sustained manner to constant current injection [18, 19]. Transient cells, such as the one shown in Fig 1A, produced sustained spike activity in response to 1s duration current as shown in Fig 1C. Increasing the strength of the current increased the number of spikes in a linear fashion. Neurons that exhibited typical transient (Fig 1A) or sustained (Fig 1B) responses to light nevertheless produced maintained spiking to a current injection. Current injection experiments in 10 transient and 5 sustained cells revealed spiking vs. current amplitude rising monotonically (Fig 1D and 1E). Thus, both transient and sustained cells generate similar prolonged and strength-dependent spike activity during maintained current injection. This implied that the transient/sustained dichotomy was produced presynaptic to the ganglion cells.

### EPSCs generated in GLCs by transient and sustained bipolar cells have different kinetics

**Fig 2:**
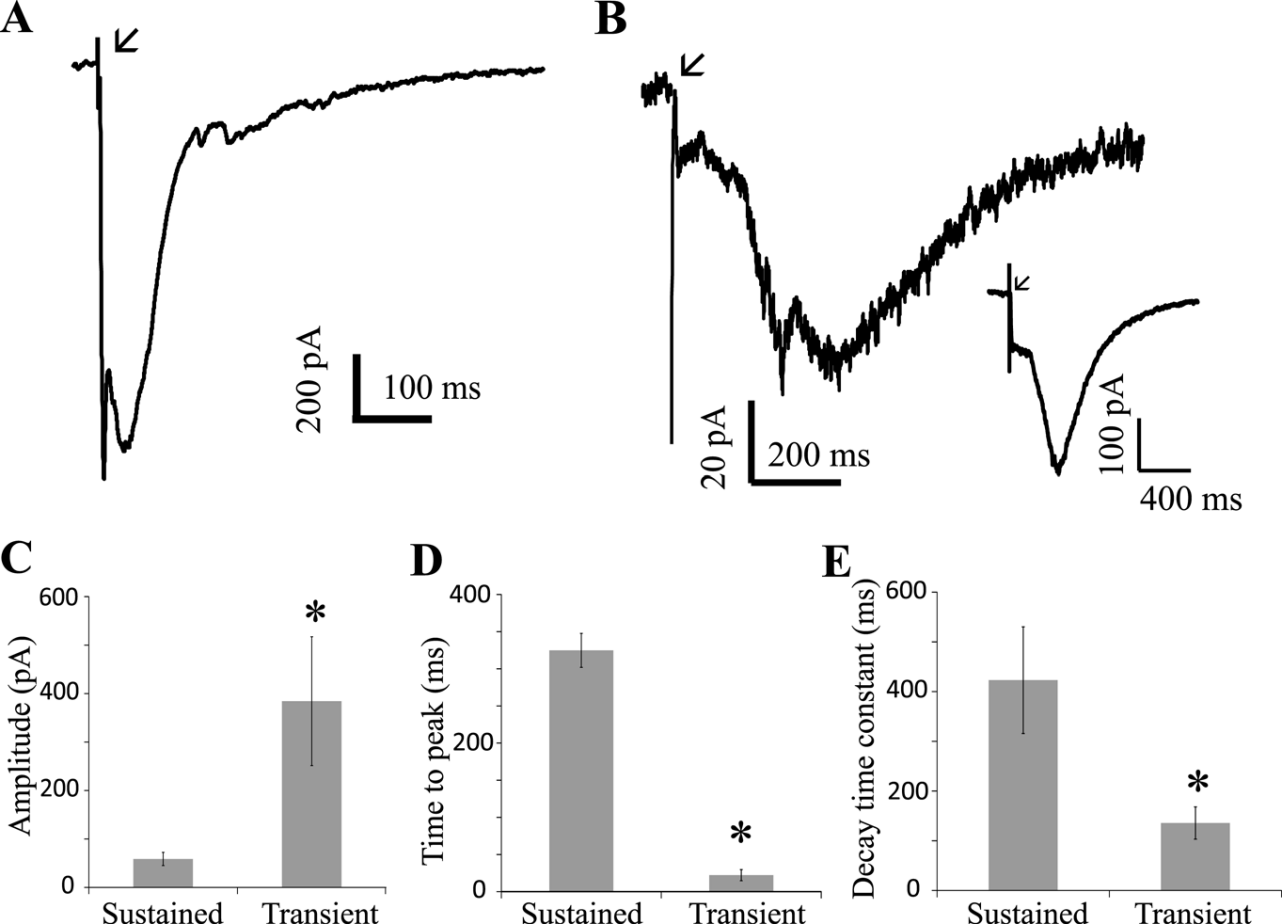
Stimulation of bipolar dendrites produces distinct EPSCs in transient and sustained GLCs. **(**A**)** and **(**B) show examples of EPSCs of a transient and sustained cell, respectively, when bipolar cell dendrites were electrically stimulated (E-EPSC) in the OPL. The E-EPSCs were recorded in PTX+STR+CTZ in Mg^2+^ free Ringer’s solution at a frequency of 0.25 Hz. The arrows indicate the time of electrical stimulus. The traces shown are an average of 10 trials. The *inset* in (B) shows the E-EPSCs of a sustained cell at a stimulus frequency of 0.1 Hz. The shape of the E-EPSC did not change with a reduction in frequency. (C) shows the comparison of the mean peak amplitude of the E-EPSCs in sustained (n = 7) and transient cells (n = 8, *p < 0.05). (D) shows the comparison of the mean time to peak amplitude of the E-EPSCs in these sustained and transient cells (*p < 0.001). (E) shows the comparison of the mean decay time constant of the L-EPSCs in these sustained and transient cells (*p < 0.05).

**Fig 3:**
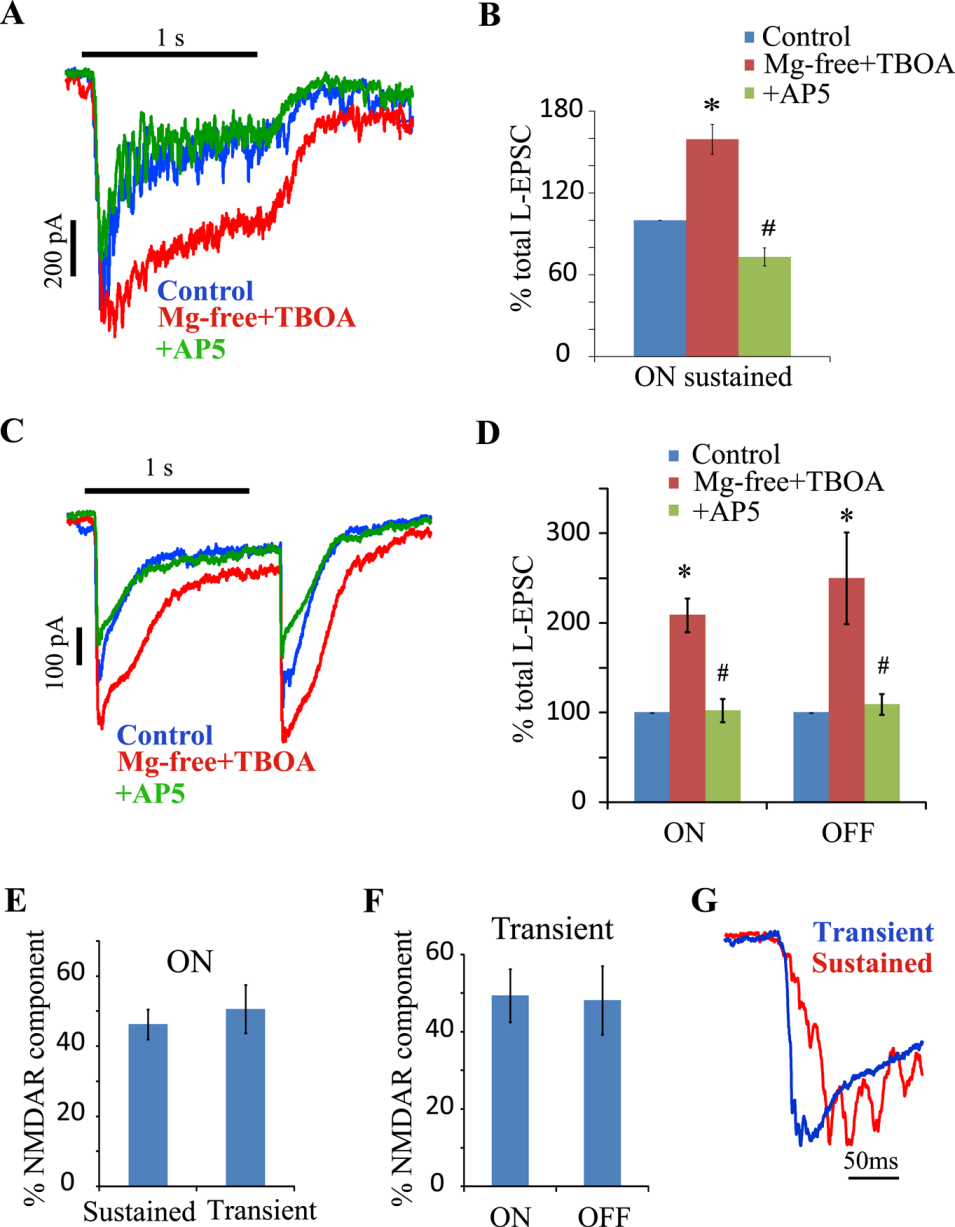
Glutamate spillover activates a similar NMDAR component in the ON L-EPSCs of both sustained and transient cells. (A) shows the L-EPSCs of a sustained cell in control, TBOA in Mg^2+^ free Ringer’s solution, and AP5 with TBOA in Mg^2+^ free Ringer’s solution. The 1s light stimulus is represented by the solid bar at the top. (B) shows a summary of the relative mean charge transferred during the L-EPSC in control, TBOA in Mg^2+^ free Ringer’s solution and AP5 with TBOA in Mg^2+^ free Ringer’s solution in ON sustained cells (n = 6). TBOA in Mg^2+^ free Ringer’s solution increased the L-EPSC compared to control (*p < 0.005) while AP5 reduced the enhanced L-EPSC (^#^p < 0.001). (C) shows the ON and OFF L-EPSCs of a transient cell in control, TBOA in Mg^2+^ free Ringer’s solution and AP5 with TBOA in Mg^2+^ free Ringer’s solution. (D) shows a summary in transient cells (n = 6) of the relative mean charge transfer during the L-EPSC in control, TBOA in Mg^2+^ free Ringer’s solution, and AP5 with TBOA in Mg^2+^ free Ringer’s solution. TBOA in Mg^2+^ free Ringer’s solution increased the L-EPSC compared to control (*p < 0.05) while AP5 reduced the enhanced L-EPSC (^#^p < 0.005). (E) compares the NMDAR component of sustained (n = 6) and transient cells (n = 6) in TBOA. The NMDAR component of the two cell types were not significantly different (p = 0.53). (F) shows a comparison of the ON and OFF transient NMDAR components (n = 6). The NMDAR components of the two responses were not significantly different (p = 0.85). (G) shows the normalized L-EPSC for the first 200ms after light onset of both transient and sustained cells in control. Sustained responses were slower to reach peak.

It has been established that subsets of bipolar cells carry transient or sustained signals based on synaptic kinetics at their dendritic inputs [2, 3]. Are there mechanisms that reinforce this dichotomy at the output synapse of bipolar cells? To test this, the salamander retinal slice preparation was used (note that this was the only section in which the retinal whole-mount retinal preparation was not used) and bipolar cell dendrites in the OPL were electrically stimulated for 1 ms at a frequency of 0.25 Hz and the electrically-evoked EPSCs (E-EPSCs) were recorded from neurons in the ganglion cell layer. This allowed evaluation of the axon terminal release properties without the influence of dendritic synaptic currents. GLCs were identified first by light-evoked extracellular spike activity and confirmed by the light evoked EPSCs in the whole cell patch clamp configuration. The E-EPSCs were recorded at -70mV in 100 μM picrotoxin (PTX), 10 μM strychnine (STR) and 30 μM cyclothiazide in Mg^2+^ free Ringer’s solution. This removed inhibitory circuits at the bipolar cell terminal, AMPAR desensitization in the postsynaptic membrane, and Mg^2+^ block of the NMDARs. Transient and sustained GLCs had different responses to the electrical stimulus (Fig 2A and 2B). Sustained cell E-EPSCs were significantly slower and smaller, as were decay time constants (Fig 2C–2E). Time to peak was 325 ± 23 ms in sustained cells and 22 ± 8 ms in transient cells. The peak amplitude was 59 ± 14 pA in sustained cells and 384 ± 133 pA in transient cells. The decay time constant was 423 ± 107 ms in sustained cells and 136 ± 32 ms in transient cells. Decreasing the frequency of stimulation to 0.1 Hz in two sustained cells increased the peak current 6.2 ± 0.5 fold but had little effect on the time to peak (1.1 ± 0.2 fold) and decay time (0.85 ± 0.29 fold), indicating that the slower synaptic signals in sustained third order cells was not due to synaptic depression (Fig 2B *inset*). Further, we were able to record both transient and sustained light-evoked responses in GLCs when stimulating the same OPL region in a retinal slice. The sustained light-evoked EPSCs were also significantly slower than transient EPSCs (time to peak was 99 ± 4 ms vs. 81 ± 2 ms after stimulus onset, respectively; n = 15, p < 0.005, Fig 3G). Thus, bipolar cells that supply transient and sustained third order neurons have discrete synaptic release properties.

### Transmitter ‘spillover’ selectively activates NMDA receptors in both sustained and transient neurons

The discrete properties of the output synapses of sustained and transient cells suggested the possibility that the glutamate receptor activation pattern in the two cell types might differ. Previous studies have shown that NMDAR localization could vary at different RGC synapses. Chen and Diamond [20] found that ganglion cell spontaneous EPSCs were produced by AMPA/KA receptors (AMPA/KARs) exclusively in rat retina, but electrically stimulated bipolar cell glutamate release activated both AMPA/KARs and NMDARs. Furthermore, the NMDAR component was enhanced when glutamate uptake was suppressed, indicative of perisynaptic NMDAR localization. Also in rat retina, Zhang and Diamond [21] localized NR2A subunits to the synapse in OFF ganglion cells, while NR2B subunits were perisynaptic at ON ganglion cells. In mouse retina, Sagdullaev et al [22] showed that loss of GABA_C_ receptors led to a decrease in sustained ON ganglion cell dynamic range and to glutamate spillover that activated NMDARs. This was not observed in sustained OFF ganglion cells. This variable influence of NMDARs raised the possibility that NMDAR activation might differ in transient and sustained ON RGC responses in amphibian retina.

The presence of perisynaptic excitatory amino acid receptors was explored by suppressing removal of synaptic glutamate using an uptake inhibitor, TBOA. In neurons clamped at -70mV, TBOA (20μM) in Mg^2+^ free Ringer’s solution increased the light stimulated synaptic currents (L-EPSCs) of both sustained (Fig 3A) and transient (Fig 3C) neurons, suggesting the presence of peri-synaptic glutamate receptors in both pathways. AP5 reduced the TBOA enhanced ON L-EPSCs in both cell types (sustained: 46 ± 4%, transient: 51 ± 7%) and returned the ON EPSCs close to their pre-TBOA levels, indicating that almost all the perisynaptic receptors were NMDARs (Fig 3B and 3D). The NMDAR current was a similar fraction of the total EPSC in transient and sustained ON light responses (Fig 3E).

TBOA also increased the OFF transient L-EPSCs (Fig 3C). AP5 reduced the OFF response to pre-TBOA levels similar to the ON transient response (OFF: 52 ± 9%, Fig 3D and 3F). In summary, the peri-synaptic receptors at both sustained ON and transient ON and OFF sites are almost exclusively NMDAR mediated and represent a similar fraction of the total synaptic current.

### Inhibition regulates release differently in transient and sustained cells

**Fig 4:**
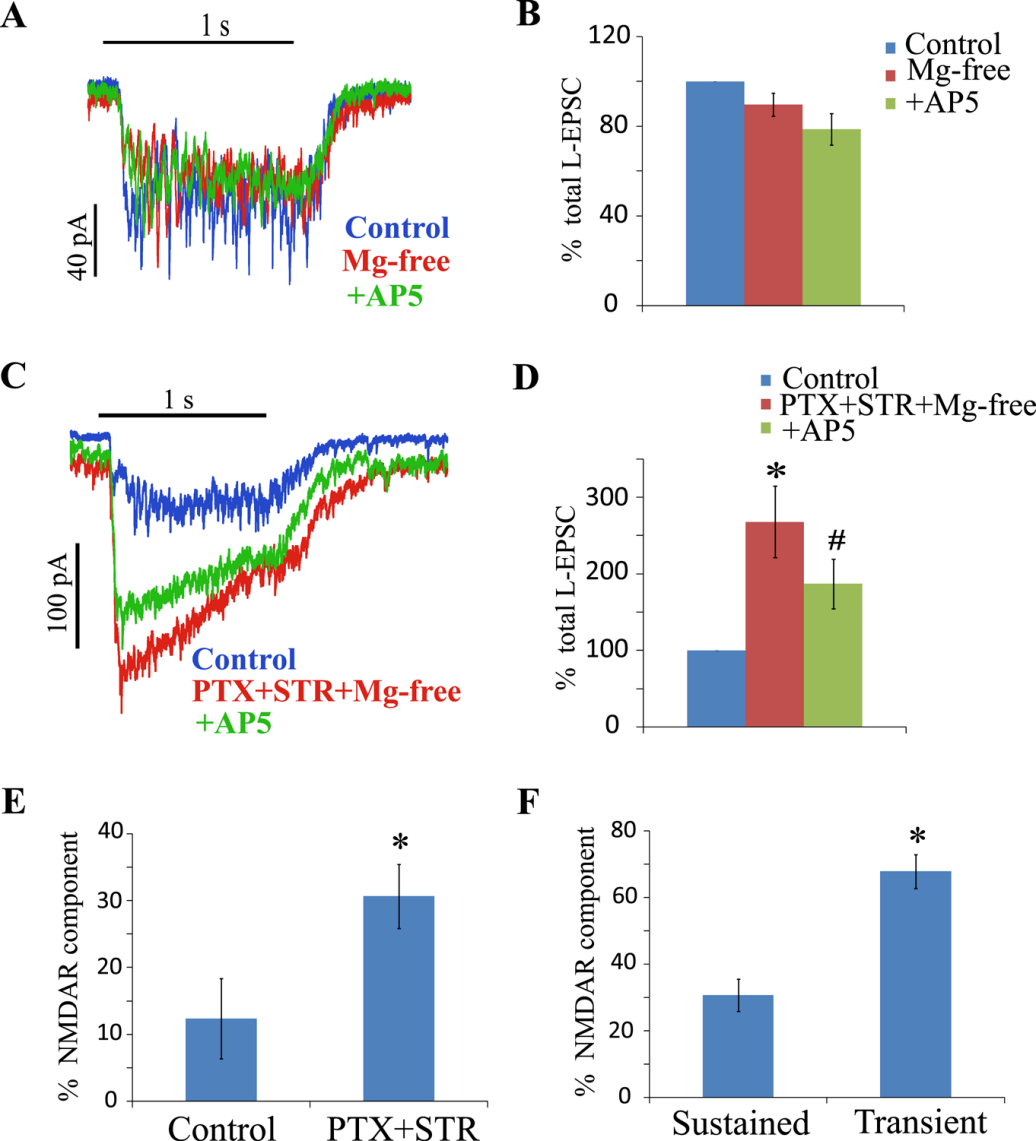
Presynaptic inhibition regulates activation of a smaller NMDAR component in sustained cells compared to transient cells. (A) shows the L-EPSCs of an ON sustained cell under control, then in Mg^2+^ free Ringer’s solution and then with addition of AP5. (B) shows the relative mean charge transfer during the sustained ON L-EPSC under control, Mg^2+^ free Ringer’s solution and plus AP5 (n = 8). Mg^2+^ free Ringer’s solution did not change the L-EPSC significantly compared to control (p = 0.09). AP5 in Mg^2+^ free Ringer’s solution did not change the L-EPSC significantly compared to Mg^2+^ free Ringer’s solution (p = 0.08). (C) shows the L-EPSCs of an ON sustained cell under control, PTX+STR in Mg^2+^ free Ringer’s solution and plus AP5. (D) shows the relative mean charge transfer during the sustained ON L-EPSC under control, PTX+STR in Mg^2+^ free Ringer’s solution and AP5 (n = 6). PTX+STR in Mg^2+^ free Ringer’s solution enhanced the L-EPSC compared to control (*p < 0.05). AP5 reduced the PTX+STR enhanced L-EPSC significantly (^#^p ≤ 0.001). (E) shows the comparison of NMDAR components in control (n = 8) and PTX+STR (n = 6). The NMÚR component in PTX+STR was higher than control (*p < 0.05). (F) shows the comparison of NMDAR components of sustained (n = 6) and transient (n = 6) cells in PTX+STR. The NMDAR component of transient cells is from our previous report [14]. The NMDAR component in transient cells is significantly higher than sustained cells (*p < 0.001).

Several studies have shown that excitatory input to transient, ON-OFF third order neurons is not blocked by NMDAR antagonists, indicating that their bipolar cell synaptic glutamate activates predominantly non-NMDARs[14, 23–28]. This is also true in sustained ON cells, as illustrated in Fig 4A and 4B. In ON cells clamped at -70 mV, a 1s light stimulus elicited a sustained inward current that was not increased when Mg^2+^ was removed from the Ringer’s solution (90 ± 5%) and was not reduced when 50 μM AP5 was added to the Ringer’s solution (88 ± 6%). However, when PTX and STR were both added to the Mg^2+^-free Ringer’s solution then the L-EPSC increased to 268 ± 47% of control amplitudes. This augmented current was partially reduced by addition of AP5 (69 ± 5% of the EPSC remained after AP5 block, Fig 4C and 4D). Thus, under these conditions, both the NMDAR and non-NMDAR currents increased. We interpret this result, coupled with the results of Fig 3 that removal of inhibition produced both spillover of glutamate to perisynaptic NMDARs and also disinhibition of additional synapses allowing non-NMDA synaptic receptors to be activated.

In previous experiments on transient ON-OFF cells [14], blocking inhibition enhanced the ON transient L-EPSC by 407 ± 72%, of which the NMDAR component was 68% (Fig 2F, [14]). The NMDA fraction of the total EPSC was twice as large in the transient (68%) compared to sustained ON response (31%, Fig 2F). Blocking inhibition produced similar increases in the non-NMDA ON EPSCs: an 85% increase in sustained cells and a 72% in transient neurons. Since spillover activated almost exclusively NMDARs, this non-NMDAR current indicates recruitment of new synapses, showing that inhibition suppresses about half of the excitatory synaptic activity under our experimental conditions.

Spillover activates a similar fraction of NMDARs in sustained and transient ON EPSCs (46% vs. 51%). Removing inhibition produces a similar fractional increase in non-NMDARs in the two cell types (85% vs. 72%), but the NMDAR current is much greater in transient EPSCs (68% vs. 31%).This suggests that there are a significantly higher number of perisynaptic NMDARs that are under inhibitory regulation in the transient pathway.

### Sensitivity and dynamic range of transient and sustained cells

**Fig 5:**
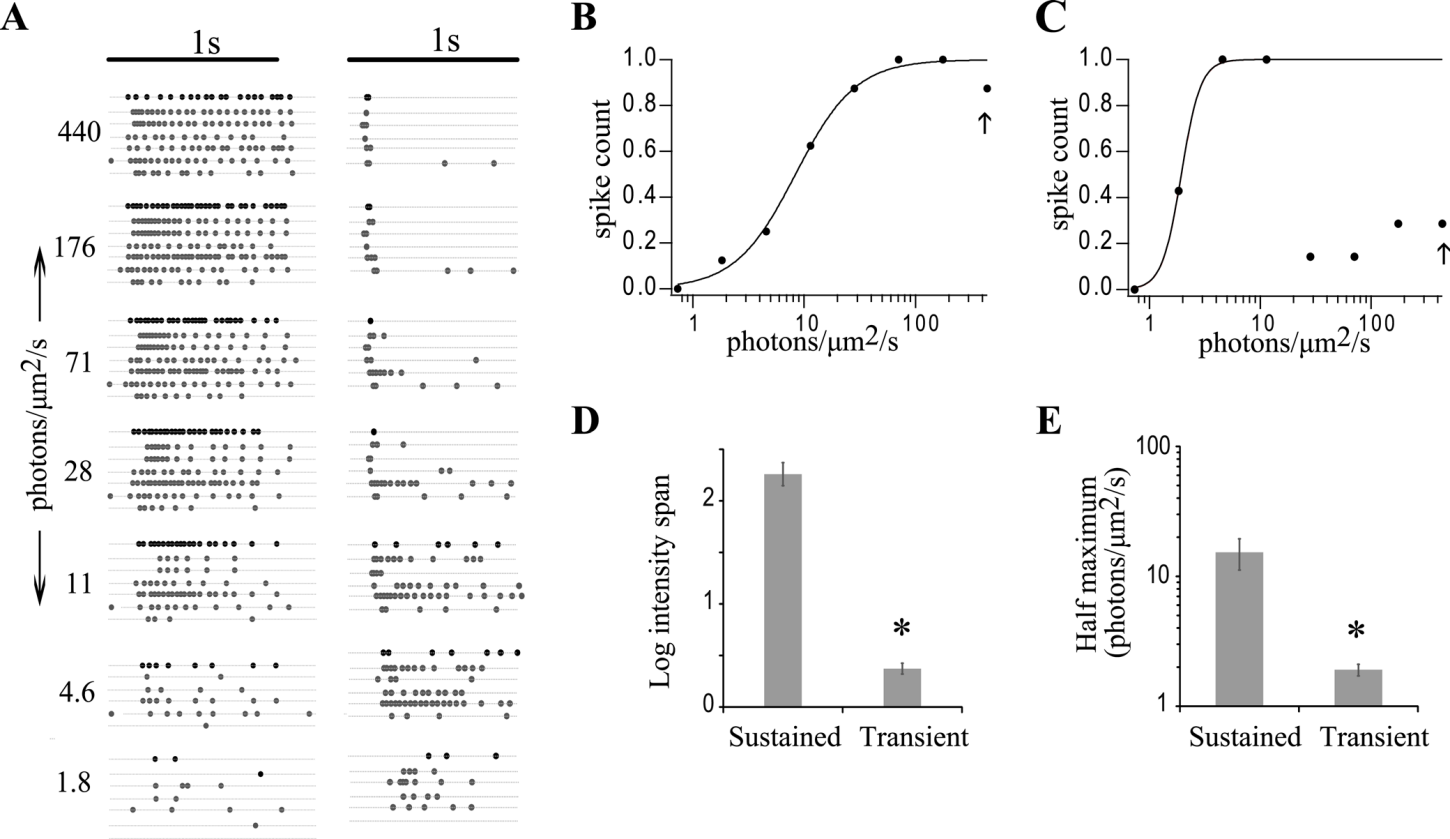
Transient and sustained GLCs encode distinct regions of the intensity spectrum. The left and right columns in (A) show the spike raster plots of sustained (n = 7) and transient cells (n = 6), respectively, to different intensities of light. (B) and (C) show the Naka-Rushton fit to normalized spike count plotted against increasing intensities of light to the sustained and transient bold responses in (A) (first trace in each intensity). The arrow indicates the light intensity used in voltage/current clamp experiments. (D) and (E) respectively show a comparison of the average intensity span and half maximum, obtained from the Naka-Rushton fits of each cell type. Averages of both intensity span and half maximum of sustained cells were significantly higher than transient cells (intensity span: *p < 0.001, half maximum: *p < 0.05).

Does this NMDAR current relate to retinal information coding? Clearly transient and sustained cells decompose visual signals differently. However, since both cell types can produce prolonged spike signals (Fig 1), their discrete roles in information coding were explored, concentrating on light intensity. ON spike responses to 1s light stimuli of various intensities were measured in sustained and transient GLCs using the loose patch technique in whole-mount retina (Fig 5A). All the transient cells recorded here were ON-OFF cells. However, for the purposes of this study we have truncated the response to show only the ON spiking. The results were fit to the Naka-Rushton equation to determine both the dynamic range and sensitivity for each cell as described in Methods (Fig 5B and 5C).

In transient cells the spike number increased up to light intensities of ~11 photons/μm^2^/s and then declined to only a few spikes at the beginning of the light stimulus (Fig 5C). The large reduction in spike activity at higher intensities was due in part to feedback inhibition and was not as pronounced in cells exposed to PTX+STR. The main effects of PTX+STR were observed at intensities after the peak spike count was reached (see below).

In contrast, ON sustained cells exhibited a monotonic increase in total spikes with increasing light intensity (Fig 5B). The total spike count in sustained cells (19.9 ± 2.2) was significantly greater than transient cells (8.4 ± 1, p < 0.001). As expected, sustained cells had a wider dynamic range (2.3 vs. 0.4 log units, Fig 5D, Table 1). However transient cells had a higher sensitivity (1.9 vs. 15.3 photons/μm^2^/s, Fig 5E, Table 1). Quantitatively, transient cells responded to only 16% of the dynamic range (span) of sustained cells but had almost an order of magnitude greater sensitivity (lower half maximum). The transient cell spike activity peaked at light levels at which sustained cells were relatively inactive. Hence the spike counts of sustained and transient cells are complementary and combine to transmit high sensitivity over their combined dynamic ranges (described below). Interestingly, transient cells changed from intensity coding (graded increase in spiking with light intensity) to a light detector (small number of spikes concentrated at light onset) at an intensity where the sustained cell intensity coding became more prominent (Fig 5A).

**Table 1:** The average half-maximum and intensity span of transient and sustained cells.

Condition	Transient cells		Sustained cells	
	Half-maximum (photons/μm^2^/s)	Intensity span (log units)	Half-maximum (photons/μm^2^/s)	Intensity span (log units)
Control	1.91 ± 0.19	0.37 ± 0.05	15.3 ± 4.12	2.26 ± 0.11
PTX+STR	2.21 ± 0.38	0.7 ± 0.13	3.47 ± 0.97	1.04 ± 0.31
PTX+STR+AP5	4.2 ± 0.73	1.35 ± 0.31	3.42 ± 0.58	1.2 ± 0.19

### Effects of inhibition and spillover on intensity coding

**Fig 6:**
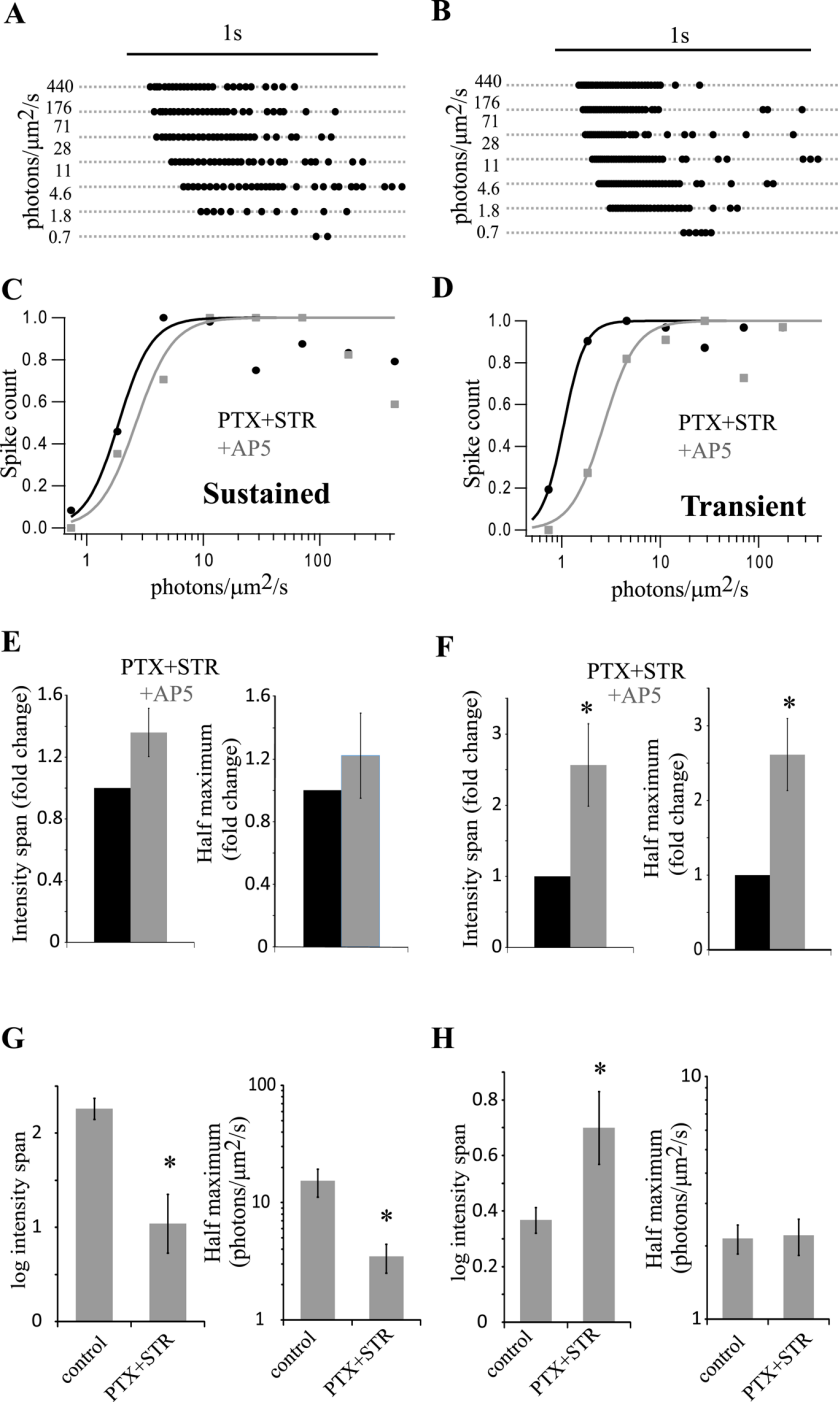
Inhibition and spillover can determine dynamic range and sensitivity. (A) and (B) respectively, show the spike raster plots of a sustained and transient cell in PTX+STR. (C) and (D) respectively, show normalized spike activity of the cells shown in (A) and (B), as a function of light intensity, in PTX+STR and PTX+STR+AP5. (E) and (F) show the average change in intensity span and half maximum in PTX+STR and PTX+STR+AP5 for transient (n = 10) and sustained cells (n = 7), respectively. Both intensity span and half maximum significantly increased in transient cells in the presence of AP5 compared to PTX+STR (span: p < 0.05*; half maximum: p < 0.01*). While, neither parameter changed in sustained cells (span: p = 0.08; half maximum: p = 0.44). (G) and (H) show the average change in intensity span and half maximum in PTX+STR compared to control in sustained and transient cells, respectively. Both intensity span and half maximum were reduced in sustained cells (span: p < 0.05*; half maximum: p < 0.05*). In transient cells, span significantly increased in PTX+STR while half maximum was unaffected (span: p < 0.05*; half maximum: p = 0.9).

Disinhibition can alter the spillover of glutamate at a synapse and also recruit new synaptic inputs. In order to investigate this dual effect of inhibition, intensity coding was evaluated in transient and sustained cells. As shown in previous literature [29], transient cells even in the absence of inhibition spiked for shorter durations of the stimulus (Fig 6A and 6B).

**Fig 7:**
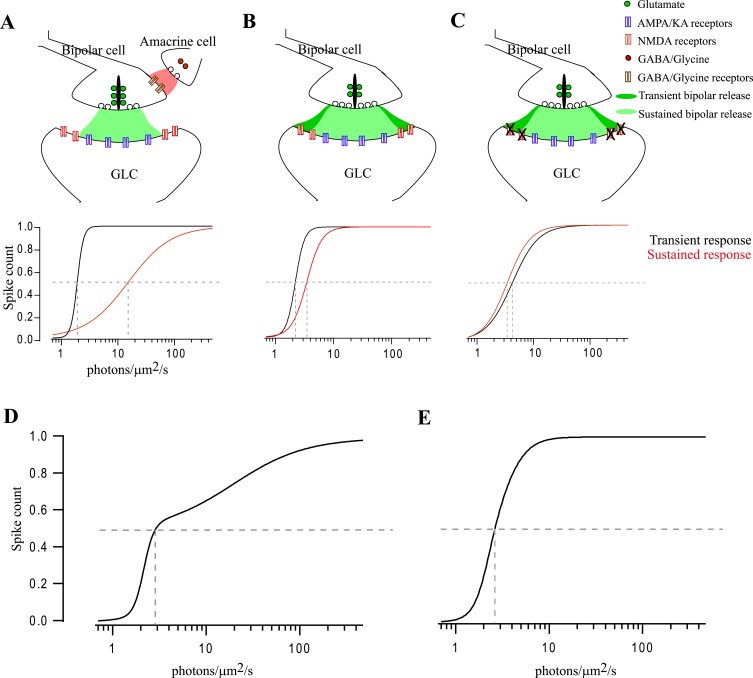
Proposed model of the bipolar-amacrine-ganglion cell synapse in encoding intensity. Ganglion layer cells express AMPA/KARs and NMDARs in the synaptic and perisynaptic space, respectively. (A) Under control conditions inhibitory circuits regulate glutamate released by both transient and sustained bipolar cell terminals, resulting in activation of predominantly AMPA/KARs. Spike count of transient cells has a small dynamic range and high sensitivity compared to sustained cells. (B) When inhibition was blocked the dynamic range and sensitivity changed, most notably for sustained cells. (C) However, the additional suppression of NMDARs had little effect on sustained cell responses but altered transient cell responses so that they carried information similar to that of sustained cells. (D) shows the combined responses of transient and sustained cells, equally weighted, under control conditions. (E) shows the combined responses of transient and sustained cells, equally weighted, when inhibition is blocked and both NMDARs and AMPA/KARs are active.

Transient ON responses recorded in PTX+STR exhibited an increased dynamic range (0.37 to 0.7 log units, p < 0.05) while sensitivity was little changed compared to control (1.9 to 2.2 photons/μm^2^/s, p = 0.9) (Fig 6D and 6H, Table 1). However, removing inhibition produced a large increase in sensitivity in sustained ON responses (15.5 to 3.5 photons/μm^2^/s, p < 0.05)while the dynamic range was reduced (2.3 to 1.0 log units, p < 0.05, Table 1, Fig 6C and 6G). In effect, blocking inhibition caused the intensity coding of sustained ON EPSCs to become more similar to transient ON EPSCs (see Table 1 and compare red traces in Fig 7A and 7B). Thus, inhibition was critical in maintaining both the low sensitivity and the large dynamic range in sustained cells.

The effects of PTX and STR could be due to spillover, recruitment of additional synapses, or indirect network effects. To test for spillover, 50 μM AP5 was applied to block NMDARs. In ON sustained cells, blocking NMDARs in the presence of PTX+STR produced a slight increase in dynamic range and a slight decrease in sensitivity, although neither effect was statistically significant (Fig 6C and 6E, Table 1). Thus, spillover does not contribute to the dynamic range of sustained cells. This is not surprising as PTX+STR had a relatively modest effect in inducing spillover as seen in L-EPSCs (Fig 4). In transient cells, PTX+STR induced a large NMDAR component in the ON L-EPSCs through spillover, suggesting a role in shaping spike responses (Fig 4,[14]). Indeed, blocking NMDARs using AP5 in the presence of PTX+STR increased the dynamic range of spike responses but reduced sensitivity (Fig 6D and 6F, Table 1).

A summary of these observations is shown in Fig 7. Under control conditions (Fig 7A) the sensitivity of the transient ON response is almost a log unit greater than the ON sustained response, but the latter has a dynamic range that is about two orders of magnitude greater. Inhibition is a key factor in generating these properties in sustained ON responses, with comparatively little influence on transient ON responses (Fig 7B). Under these conditions, there is little difference in intensity coding of sustained and transient cells and the net output of the retina has reduced sensitivity and dynamic range (Fig 7D and 7E). NMDARs presumptively from spillover, has little effect on the sustained cell intensity coding, but help transient cells in maintaining their response characteristics (Fig 7B and 7C).

## Discussion

This study explored factors at the second order retinal synapse that contribute to generation of transient and sustained ON signals. Differences in both the bipolar terminals and the RGC itself were studied. Both transient and sustained RGCs expressed AMPA/KARs synaptically and NMDARs perisynaptically and hence activation of NMDARs was a reporter for spillover of glutamate. Blocking feedback inhibition increased the NMDAR activation in both cell types but was more extensive in transient cells. This difference in NMDAR activation may be explained by the bipolar release kinetics. When spike outputs were observed, transient and sustained RGCs exhibited linear increases in spike activity to injected current. But, for light stimuli, spiking in transient RGCs had higher sensitivity and smaller dynamic range compared to sustained RGCs. Transient but not sustained cells maintained their features when feedback inhibition was blocked and this was made possible by induction of NMDAR activation.

### Coding Intensity

Transient and sustained ganglion cells are characterized by their responses to moderate or bright stimuli. However, even transient neurons respond with prolonged spiking to a dim light (Fig 5) and the spike count is correlated with intensity. Furthermore, the prolonged response in transient cells fades as the intensity coding in sustained cells becomes robust. Sustained cells have a lower sensitivity, as measured by half-maximum response, and larger dynamic range. But this is dependent on inhibition. Without inhibition sustained cell sensitivity and dynamic range approach values of transient cells (Table 1 and Fig 7). Inhibition has comparatively little effect on these properties in transient cells and this is likely to be due to the activation of peri-synaptic NMDARs.

Some of the spike coding differences could be due to intrinsic variability in the two cell types. Studies have shown that subtypes of RGCs differ in their spike patterns due to differences in voltage gated channels and also possibly due to morphology [30–34]. These intrinsic differences might play a significant role in shaping light evoked spiking under some circumstances. However, for the purpose of spike responses studied here, both cell types generated linear increases in spike number with increases in current strength. Hence the differences generated in the two pathways are mostly pre-synaptic to the third order neurons.

### Distinct release of glutamate at bipolar terminals

Transient and sustained ganglion cells in salamander retina receive inputs from distinct bipolar cell types [3]. These labeled lines are seen in other cell types, such as the ONα-like ganglion cell in the mouse retina where non-linear properties are imparted by distinct bipolar cells [35]. In the transient/sustained pathway, the bipolar cells feeding transient ganglion cells have phasic EPSCs while sustained bipolar cells have tonic light responses and the distinctions persist in the absence of feedback inhibition [3, 29]. Since the photoreceptor inputs to the two types of bipolar cells were presumably the same, it indicated that the synaptic mechanisms at the bipolar cell dendrites differed. Evidence for such mechanisms has been provided in the OFF bipolar cells where discrete activation of AMPA and KA receptors generates different temporal responses [2]. The extracellular current stimulation experiments indicate that the release properties at the axon terminals of sustained and transient bipolar cells also differ. When a 1ms electrical pulses stimulated bipolar cells, the outputs to sustained third order neurons were more prolonged, lower in amplitude and slower to rise and decay. This distinction could be achieved by different desensitization kinetics of post-synaptic AMPA receptors. For instance, a fast de-sensitizing AMPA receptor could truncate the post-synaptic membrane current and thus produce a transient response while a non-desensitizing receptor would produce a sustained response. The retina expresses both types of receptors [20, 36, 37]. However, our recordings were obtained after blocking AMPAR desensitization. Yet another possibility is that the stimulation technique might not activate enough sustained bipolar cells compared to transient cells as their densities might be different. Indeed bipolar cell densities in mouse retina have been shown to be different among subtypes [13]. However, the slowness of the E-EPSCs is not an artifact of the stimulation technique as sustained L-EPSCs were also slower to reach peak. Hence the output signals from sustained bipolar cells are intrinsically longer in duration compared to transient cells. Indeed, recent work has shown that bipolar cell subtypes have intrinsic differences in voltage gated channels resulting in differences in temporal coding of transient and sustained cells [38–42]. Even the volume of the bipolar cell terminal might play a role in determining their temporal signaling [43].

The kinetics of L-EPSCs are modulated by pre-synaptic inhibitory circuits and by the post-synaptic glutamate receptors [44–47]. NMDARs are found at OFF, but not ON, synapses in mammalian retina [21, 22]. In the guinea pig retina exogenous application of NMDA induced currents in OFFα and OFFδ ganglion cells but not in ONα cells [48]. NMDARs are often found to be located in the perisynaptic space and hence might have specific requirements for activation [20, 49, 50]. Presynaptic inhibition plays a significant role in spillover and NMDAR activation [22]. Our findings extend this model to ON synapses of both transient and sustained cells, where AMPA/KARs are synaptic while NMDARs are predominantly perisynaptic (Fig 7). Interestingly, the fractional NMDAR components of L-EPSCs, induced by TBOA, were similar in sustained and transient neurons (46 vs. 51%, respectively). So were the fractional non-NMDAR populations exposed by disinhibition (85% vs. 72%, respectively). However, while disinhibition exposes a similar fraction of new AMPA/KAR synapses, the relative population of NMDAR is much greater in transient cells (65% vs. 31%). Hence inhibition regulates not only the volume of glutamate release at the synapse [22] but also activation of additional synapses. Comparing the effects of disinhibition and uptake-suppression suggests that inhibition does not produce the maximal spread of glutamate at sustained ON synapses. The same comparison in transient cells might indicate that the additional synapses recruited by disinhibition have a larger perisynaptic NMDAR population.

### AMPA/KARs enhance dynamic range, NMDARs enhance sensitivity

Reports suggest that AMPA/KARs and NMDARs complement each other in extending dynamic range of ganglion cells [22, 51–53]. Our results show a reduction in dynamic range when both NMDARs and AMPA/KARs are active compared to AMPA/KARs alone. While dynamic range in ON sustained cells was not significantly altered by NMDAR block, it was dramatically reduced by removing inhibition.

AMPA/KARs are utilized to extend range of encoding intensity. Evidence for this is threefold. 1) Sustained cells, which had a higher dynamic range, used a smaller NMDAR component (in PTX+STR). 2) Blocking NMDARs in transient cells increased the dynamic range while still generating the same number of spikes (PTX+STR: 18 ± 1.7 spikes; AP5+PTX+STR: 17 ± 2.4 spikes). 3) Blocking NMDARs in sustained cells did not significantly affect dynamic range. It is possible that sustained cells utilize AMPA/KARs because they saturate at higher levels of synaptic glutamate thus providing the ability to respond to more intensity levels [54, 55].

Unlike dynamic range, sensitivity in the presence of both AMPA/KARs and NMDARs was higher than AMPA/KARs alone. This is particularly notable in transient cells, which have the highest sensitivity. This suggests that NMDARs can significantly increase the sensitivity of visual information sent from the retina. In support of this role, it has been noted that NMDARs relay low contrast signals in select cell types [48, 53].
